# Determinants of photochemical characteristics of the photosynthetic electron transport chain of maize

**DOI:** 10.3389/fpls.2023.1279963

**Published:** 2023-11-20

**Authors:** Xiuping Liu, Yunzhou Qiao, Wangming Zhou, Wenxu Dong, Lianhong Gu

**Affiliations:** ^1^ Key Laboratory of Agricultural Water Resources, Hebei Key Laboratory of Soil Ecology, Center for Agricultural Resources Research, Institute of Genetics and Developmental Biology, Chinese Academy of Sciences, Shijiazhuang, China; ^2^ School of Life Sciences, Anqing Normal University, Anqing, China; ^3^ Environmental Sciences Division and Climate Change Science Institute, Oak Ridge National Laboratory, Oak Ridge, TN, United States

**Keywords:** photosynthesis, photosynthetic electron transport, redox parameters, leaf characteristics, plant growth stages, maize

## Abstract

**Introduction:**

The photosynthetic electron transport chain (ETC) is the bridge that links energy harvesting during the photophysical reactions at one end and energy consumption during the biochemical reactions at the other. Its functioning is thus fundamental for the proper balance between energy supply and demand in photosynthesis. Currently, there is a lack of understanding regarding how the structural properties of the ETC are affected by nutrient availability and plant developmental stages, which is a major roadblock to comprehensive modeling of photosynthesis.

**Methods:**

Redox parameters reflect the structural controls of ETC on the photochemical reactions and electron transport. We conducted joint measurements of chlorophyll fluorescence (ChlF) and gas exchange under systematically varying environmental conditions and growth stages of maize and sampled foliar nutrient contents. We utilized the recently developed steady-state photochemical model to infer redox parameters of electron transport from these measurements.

**Results and discussion:**

We found that the inferred values of these photochemical redox parameters varied with leaf macronutrient content. These variations may be caused either directly by these nutrients being components of protein complexes on the ETC or indirectly by their impacts on the structural integrity of the thylakoid and feedback from the biochemical reactions. Also, the redox parameters varied with plant morphology and developmental stage, reflecting seasonal changes in the structural properties of the ETC. Our findings will facilitate the parameterization and simulation of complete models of photosynthesis.

## 1 Introduction

The photosynthetic electron transport chain (ETC) between photosystem II (PSII) and photosystem I (PSI) is a key bridge in photosynthesis. It links photophysical reactions at one end and biochemical reactions at the other, while photochemical reactions occur on this bridge (Kamen, 1963; Gu et al., 2023a). The photophysical reactions are responsible for photon harvesting, exciton transfer to reaction centers, and the dissipation of excess energy as heat and fluorescence. The photochemical reactions convert the excitons into free electrons and transport them down the ETC, resulting in water splitting in the lumen, proton translocation from the stroma to the lumen, and the synthesis of NADPH and ATP. The biochemical reactions use the NADPH and ATP produced to reduce CO_2_ to sugars and to oxygenate RuBP (Kamen, 1963; Gu et al., 2023a). Because the three stages of the reactions are sequentially connected, the balance between them is crucial for the photosynthetic machinery to operate productively and safely in fluctuating environments.

Among the three reaction stages of photosynthesis at the leaf scale, the photochemical reactions at the ETC are the least studied. The photophysical reactions have long been probed with Pulse Amplitude-Modulated (PAM) fluorometry (Baker, 2008), whereas extensive research has been conducted on the biochemical reactions with the gas exchange technique (von Caemmerer and Farquhar, 1981). In contrast, there has been a lack of a technique that can be used to directly and quantitatively monitor the ETC and the photochemical reactions that occur on it. This lack of quantitative study of the ETC and the photochemical reactions has hindered progress on multiple fronts. For example, many components of the photophysical and biochemical reactions have been targeted for bioengineered modification to improve the efficiency of photosynthesis, including the size of the light-harvesting antenna complex (Kirst et al., 2017; Cardona et al., 2018), nonphotochemical quenching (Kromdijk et al., 2016; De Souza et al., 2022), and Rubisco kinetics (Lin et al., 2014). Currently, it is not clear whether the ETC will be capable of supporting these proposed modifications in such a way that the modified photosynthetic machinery will operate as expected under field conditions. This issue is of relevance for crop bioengineering because the ETC is likely suboptimal under current environmental conditions (Chida et al., 2007; Simkin et al., 2017; Ermakova et al., 2019) and may also need to be modified to support increased electron transport demand without suffering from excessive photooxidative stress (Sonoike, 2011; Vass, 2012). Resolving this issue will require a better understanding of the ETC and the factors that control photochemical reactions (Gu, 2023b).

Another example of areas that call for increased research on the ETC is applications of sun-induced chlorophyll fluorescence (SIF) to monitor CO_2_ assimilation in real time under field conditions (Sun et al., 2023a; Sun et al., 2023b). SIF is emitted during the photophysical reactions as one of the dissipation pathways of photon energy harvested by antenna complexes, while CO_2_ assimilation is part of the biochemical reactions. Using SIF to infer CO_2_ assimilation requires a complete model of photosynthesis that integrates the photophysical, photochemical, and biochemical reactions. To make this complete model of photosynthesis a reality, knowledge of electron transport along the ETC is essential (Gu et al., 2019).

Recently, we developed a mechanistic photochemical model of electron transport that relates the rate of linear electron transport to the redox state of PSII (Gu et al., 2023a). This model can be coupled with the photophysical model of Gu et al. (2019) and the biochemical model of Farquhar et al. (1980) to form a complete model of photosynthesis. However, before such a complete model of photosynthesis can be applied, it is necessary to determine the variabilities of the parameters of the photochemical model and which biotic and abiotic factors may affect these variabilities. This is analogous to the study of parameter variabilities in the biochemical model of photosynthesis (*e.g.*, Walker et al., 2014). The parameters of the photochemical model reflect the characteristics of the redox reactions, which are controlled by the structural properties of the ETC. In this study, we conducted PAM fluorometry and gas exchange measurements on maize leaves at different developmental stages and canopy depths. After these measurements, leaf samples were taken for laboratory analyses of nutrient content and morphology. We inferred photochemical parameters from PAM fluorometry measurements and related them to foliar nutrient content and morphological indices to answer the following questions: How variable are the photochemical parameters of the ETC? Are photochemical parameters affected by leaf nutrient contents and morphology? How are variations in photochemical parameters related to each other? As the first study in this area, the answers to these questions should guide future research on other species.

## 2 Materials and methods

### 2.1 Site description and plant material

The study was conducted at the Luancheng Agro-ecological Experimental Station (37°53′ N, 114°41′ E, elevation 50.1 m). The dominant cropping system in this region is winter wheat (*Triticum aestivum* L.) and summer maize (*Zea mays* L.) rotations without fallow. Summer maize is planted from mid-June to early October, and winter wheat is cultivated from late October to early June of the following year. The climate is a warm temperate semi-humid monsoon, with cold winters and warm summers. The mean annual air temperature and precipitation for the period 1984 - 2016 were 12.6°C and 438.3 mm, and in 2022, 25.3°C and 492 mm occurred in the summer maize season (June-September), respectively. The soils are mainly cinnamon soils, containing 23.4 g kg^-1^ organic matter, 1.42 g kg^-1^ total N, 135 mg kg^-1^ available N, 18.9 mg kg^-1^ available P, and 98.4 mg kg^-1^ available K in the topsoil (0-20 cm).

Summer maize, Zhengdan 958, was sown on 18 June 2022 at 60 cm row spacing and 25 cm plant spacing, and harvested on 4 October 2022. Before sowing, winter wheat was harvested, and the straw was chopped and returned to the field. Subsequently, summer maize was planted with a no-till seeder equipped with a fertilizer distributor, and 600 kg ha^-1^ compound fertilizer (N: P_2_O_5_: K_2_O = 30:5:5) was applied as basal fertilizer. On the fifth day after sowing, the plants were fully irrigated to ensure even germination. With the exception of a herbicide application in early July, no other management measures were applied during plant growth.

### 2.2 Gas exchange and chlorophyll fluorescence measurements

Using fully expanded leaves from the bottom to the top of the canopy, combined gas exchange and chlorophyll fluorescence (ChlF) measurements of summer maize were conducted at the seedling (8 July), jointing (17 July), flowering (29 July, 11 August), filling (20 August), and maturity (7 September, 21 September) stages. Measurements were made between 9:00 and 16:00 using a LI-6400XT portable photosynthesis system (Li-Cor, Lincoln, NE, USA) fitted with a LI-6400-40 fluorescence leaf chamber. It is important to note that photosynthetic light response and CO_2_ response curves were measured together with ChlF on one leaf without moving the leaf chamber in between. Minimum fluorescence (*F_o_*) and maximum fluorescence (*F_m_*) in the dark-adapted state were measured pre-dawn. To produce light response curves and ChlF, the portable photosynthesis system was set to 400 μmol mol^-1^ CO_2_, airflow at 500 μmol s^-1^, photosynthetically active radiation (*PAR*) at 1800 μmol m^-2^ s^-1^, and leaf temperature and relative humidity at ambient levels. Once net photosynthesis rate (*A*) and stomatal conductance (*g_s_*) were stable, *PAR* was lowered sequentially to 1600, 1400, 1200, 1000, 800, 600, 400, 200, 150, 100, 50, 20, and 0 μmol m^-2^ s^-1^, and measurements were taken at each *PAR* as *A* reached steady state. Following completion of the light response curves, *PAR* was switched to 1600 μmol m^-2^ s^-1^, and CO_2_ response curves and ChlF were measured using the following sequence of CO_2_ concentrations: 400, 300, 200, 150, 100, 50, 400, 400, 600, 800, 1000, and 1200 μmol mol^-1^. Before each measurement, the CO_2_ and water vapor concentrations were automatically matched between the leaf and reference chambers. Finally, a total of 22 ChlF and gas exchange data were obtained for parameter calculation.

### 2.3 Leaf characteristics

After gas exchange measurements, leaves were harvested for the determination of leaf area with an electronic area meter (LI-3000A, Li-Cor, Lincoln, NE), leaf thickness with a digital micrometer (Mitutoyo, Japan), and dry mass after drying at 60°C to a constant mass. Specific leaf weight was then calculated as the ratio of leaf dry weight to leaf area. Subsequently, dried leaf samples were ground, nitrogen content (N) was determined by the Kjeldahl method (Kjeltec 8400, Foss, Sweden), phosphorus content (P) was measured by the molybdate colorimetric method (UV-2450, Shimadzu, Japan), and potassium (K) and calcium (Ca) contents were determined by a flame atomic absorption spectrophotometer (Analytik Jena, Germany). In total, six leaf characteristics were quantified: leaf thickness, specific leaf weight, foliar N, P, K, and Ca contents.

### 2.4 Inference of redox reaction parameters

Gu et al. (2023a) derived a steady-state redox model to study the relationship between electron transport and the redox state of the ETC. According to this model, the linear electron transport (LET) rate from PSII to PSI (*J_PSII_*) is photochemically related to the fraction of open PSII reaction centers (*q*) via the following equations:


(1) $$J_{PSII}=\frac{2Uf_{T}f_{s}f_{q}(q_{r}-q)q}{(R_{1}+2R_{2}f_{s}f_{q}-1)q+q_{r}},$$

(2) $$U=uN_{PQ_{T}}N_{cyt_{T}},$$

(3) $$R_{1}=\frac{r_{r}}{r_{d}},$$

(4) $$R_{2}=\frac{u}{r_{d}}\times \frac{N_{cyt_{T}}}{N_{PSII}},$$

(5) $$f_{T}=\sqrt{\frac{T_{0}}{T}}e^{E_{T}(\frac{1}{T_{0}}-\frac{1}{T})},$$

(6) $$f_{s}=\frac{v}{v_{max}}=\frac{1}{1+c_{s}e^{-b_{s}\times \alpha PAR}},$$

(7) $$f_{q}=\frac{1+a_{q}}{1+a_{q}\times q},$$

where *U* is the maximum oxidation potential of the combined mobile plastoquinone/plastoquinol (PQ/PQH_2_) pool by the cytochrome b_6_f complex (Cyt). *R_1_* and *R_2_* are the first and second electron transport resistances, respectively. *u* is the second-order rate constant for the oxidation of PQH_2_ by the RieskeFeS protein of Cyt. *r_d_* and *r_r_* are the second-order rate constants for the electron transfer from the reduced acceptor to PQ to form PQH_2_ and for the reverse reaction, respectively. $N_{PSII}$ , $N_{PQ_{T}}$ , and $N_{cyt_{T}}$ are the total foliar concentrations of PSII, the combined PQ and PQH_2_ pool, and Cyt for linear electron transport, respectively. *q_r_* is the fraction of reversible PSII reaction centers, which may be less than unity due to the presence of inhibited and Q_B_-nonreducing PSII reaction centers and the two-electron gate.

Equation 1 also contains three function modifiers, *f_T_*, *f_s_*, and *f_q_*. *f_T_* (Equation 5) is the standardized temperature (*T*) response function for modifying redox reactions derived from the Marcus theory of electron transfer in proteins. *E_T_* is a composite temperature sensitivity parameter related to the Gibbs free energy of activation. *T*_0_ = 298.15 K is the reference temperature. *f_s_* (Equation 6) is the light-induced thylakoid ultrastructure dynamic function, which quantifies the degree of thylakoid ultrastructural control over electron transport. This ultrastructural control is achieved by regulating the effect of macromolecular crowding on the diffusion of mobile electron carriers and the effective availability of Cyt for linear electron transport (LET) (Gu et al., 2022). *v* is the total thylakoid volume at a given *PAR* level and swells/shrinks in response to osmotic water fluxes into and out of the lumen, similar to the guard cell turgor pressure dynamics. *v_max_* is the maximum thylakoid volume when it is fully swollen. *b_s_* controls the speed of light-induced swelling/shrinking, while *c_s_* inversely determines the maximum net impact of macromolecular crowding on the effective availability of Cyt for LET. *f_s_* varies between a value determined by *c_s_* (thylakoid minimally shrunk in the dark) and 1 (thylakoid maximally expanded in full light). *f_q_* is the photosynthetically controlled redox poise balance function between Cyt and PSII, with *a_q_* as the redox poise stoichiometry parameter. This function relates the fraction of Cyt available for LET, denoted by *h_Cyt_*, to the fraction of PSII open reaction centers (*i.e.*, *q*) via *h_Cyt_* = *f_q_* × *q*. *a_q_* = 0 gives the redox isocline between Cyt and PSII (*h_Cyt_* = *q*, Gu et al., 2023a), and is a special case that has been assumed in previous studies (*e.g.*, Johnson and Berry, 2021). If *a_q_* > 0, *h_Cyt_* > *q*, it indicates that PSII is more strained than Cyt for LET. If *a_q_* < 0, *h_Cyt_* < *q*, it indicates that Cyt is more strained than PSII for LET.

It should be noted that the photochemical model of the *J_PSII_* – *q* relationship of Gu et al. (2023a) is complementary to, but fundamentally different from, the corresponding photophysical *J_PSII_*- *q* relationship of Gu et al. (2019). The former is a consequence of redox reactions at the ETC, while the latter reflects the partitioning of the absorbed energy into different dissipation pathways according to the principle of energy conservation. These two models can be used together to couple the photophysics with the photochemistry of photosynthesis. A detailed discussion of this topic is available in Gu et al. (2023a).

Using the steady-state photochemical model in conjunction with ChlF and gas exchange data, we can infer the redox parameters of summer maize at different growth stages. To do so, we calculated *J_PSII_* (μmol m^-2^ s^-1^) with $J_{PSII}=\Phi_{PSII}\times \alpha \beta PAR$ . Here, $\Phi_{PSII}=1-\frac{F_{s}}{F_{m}^{'}}$ is the photochemical yield of PSII, *PAR* (μmol m^-2^ s^-1^) is the incident photosynthetically active radiation, α = 0.85 is the leaf absorptance in PAR , and β = 0.5 is the fraction of absorbed *PAR* allocated to PSII. We assumed a lake model of photosynthetic unit connectivity and calculated *q* as $q_{l}=\frac{F_{m}^{'}-F_{s}}{F_{m}^{'}-F_{o}^{'}}\times \frac{F_{o}^{'}}{F_{s}}$ (Kramer et al., 2004), $F_{o}^{'}=\frac{F_{o}}{\frac{F_{v}}{F_{m}}+\frac{F_{o}}{F_{m}^{'}}}$ (Oxborough and Baker, 1997) and $F_{m}^{'}$ are minimum, maximum fluorescence yield in the light-adapted state, and *F_o_* and *F_m_* are minimum, maximum fluorescence yield in the dark-adapted state, respectively, and *F_s_* is the steady-state fluorescence yield. As explained in Gu et al. (2023a), the form of Equation 1 is independent of any assumption regarding the connectivity of the photosynthetic unit, but its parameter values may depend on whether the lake or puddle model is assumed, as the value of *q* (but not $\Phi_{PSII}$ and therefore *J_PSII_*, Kramer et al., 2004) will be different.

Equation 1 contains eight independent composite redox parameters in total (*U*, *R_1_*, *R_2_*, *q_r_*, *E_T_*, *c_s_*, *b_s_*, and *a_q_*). We estimated these eight redox reaction parameters by fitting the modeled *J_PSII_* as closely as possible to the measured values with the Tool for Optimizing the Open-Closed Redox Model (TOOCRM) of photosynthetic electron transport developed by and provided in Gu et al. (2023a). TOOCRM is a convenient Excel spreadsheet-based tool that uses the evolutionary method for parameter optimization.

### 2.5 Statistical analyses

We evaluated model performance by comparing predicted and measured *J_PSII_* – *q* relationships at different growth stages and canopy heights and examining their correlation. The relationships between redox parameters (*U*, *R_1_*, *R_2_*, *q_r_*, *a_q_*, *E_T_*, *b_s_*, and *c_s_*) and leaf characteristics (specific leaf weight, leaf thickness, N, P, K, and Ca), in addition to the relationships among these redox parameters, were examined by multiple regression. The goodness of fit of the model was evaluated by the coefficient of determination (R^2^) and analysis of variance (*p*-value). Multivariate partial least squares regression, including leave-one-out cross-validation and jackknife estimation of regression coefficients, was performed to examine the associations between redox parameters and specific leaf weight, leaf thickness, N, P, K, and Ca. Additionally, we analyzed the temporal variation of redox parameters and leaf characteristics at different growth stages.

## 3 Results

### 3.1 Model performance

**Figure 1:**
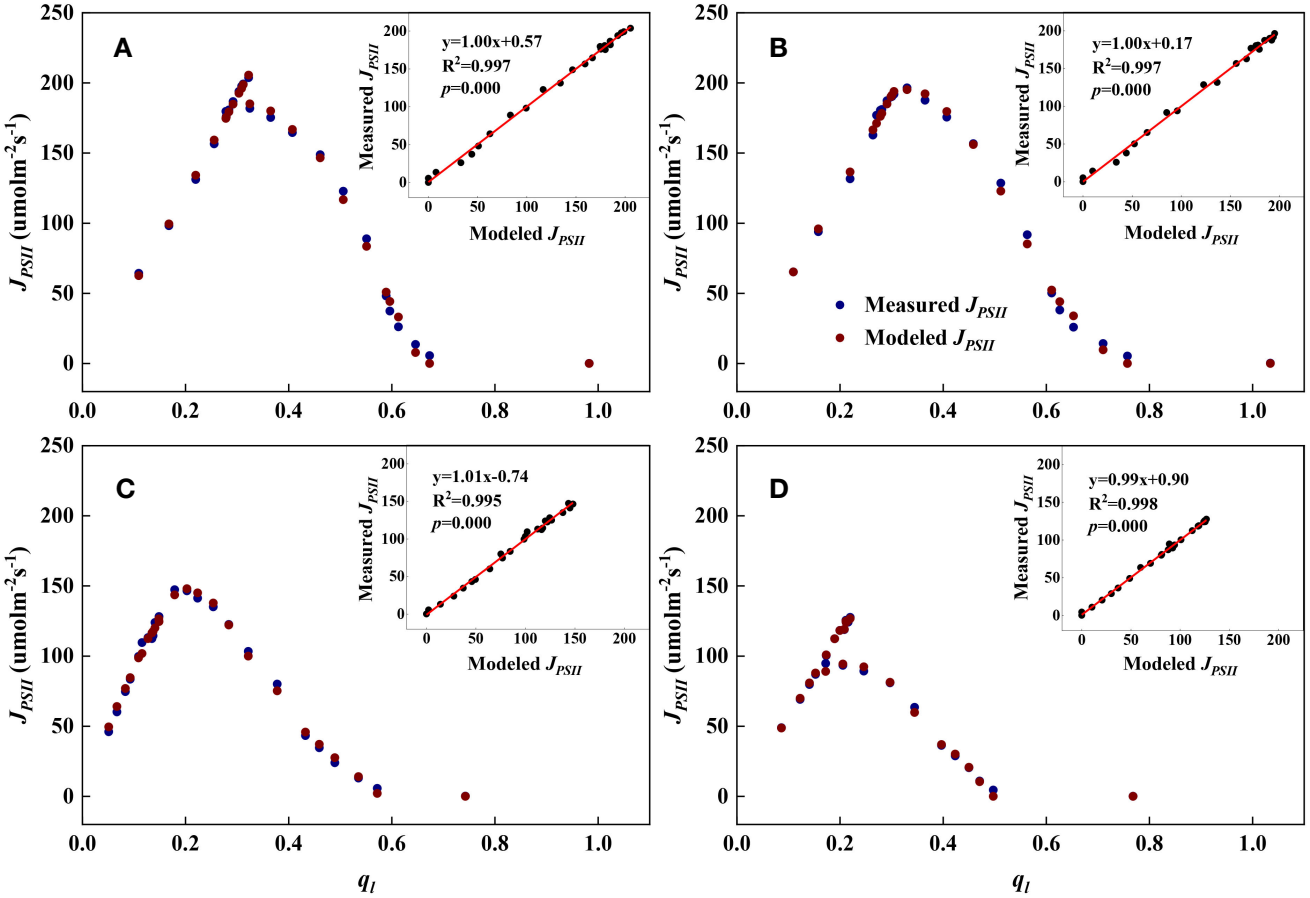
Examples demonstrating the performance of the steady-state photochemical model for predicting the linear electron transport rate (*J_PSII_*) as a function of the fraction of open PSII reaction centers (*q_l_*) for top canopy leaves at different growth stages. Inset: Comparison of measured vs. modeled *J_PSII_*. **(A)**: Jointing (17 July); **(B)**: Flowering (29 July); **(C)**: Filling (20 August); **(D)**: Maturity (7 September).

**Figure 2:**
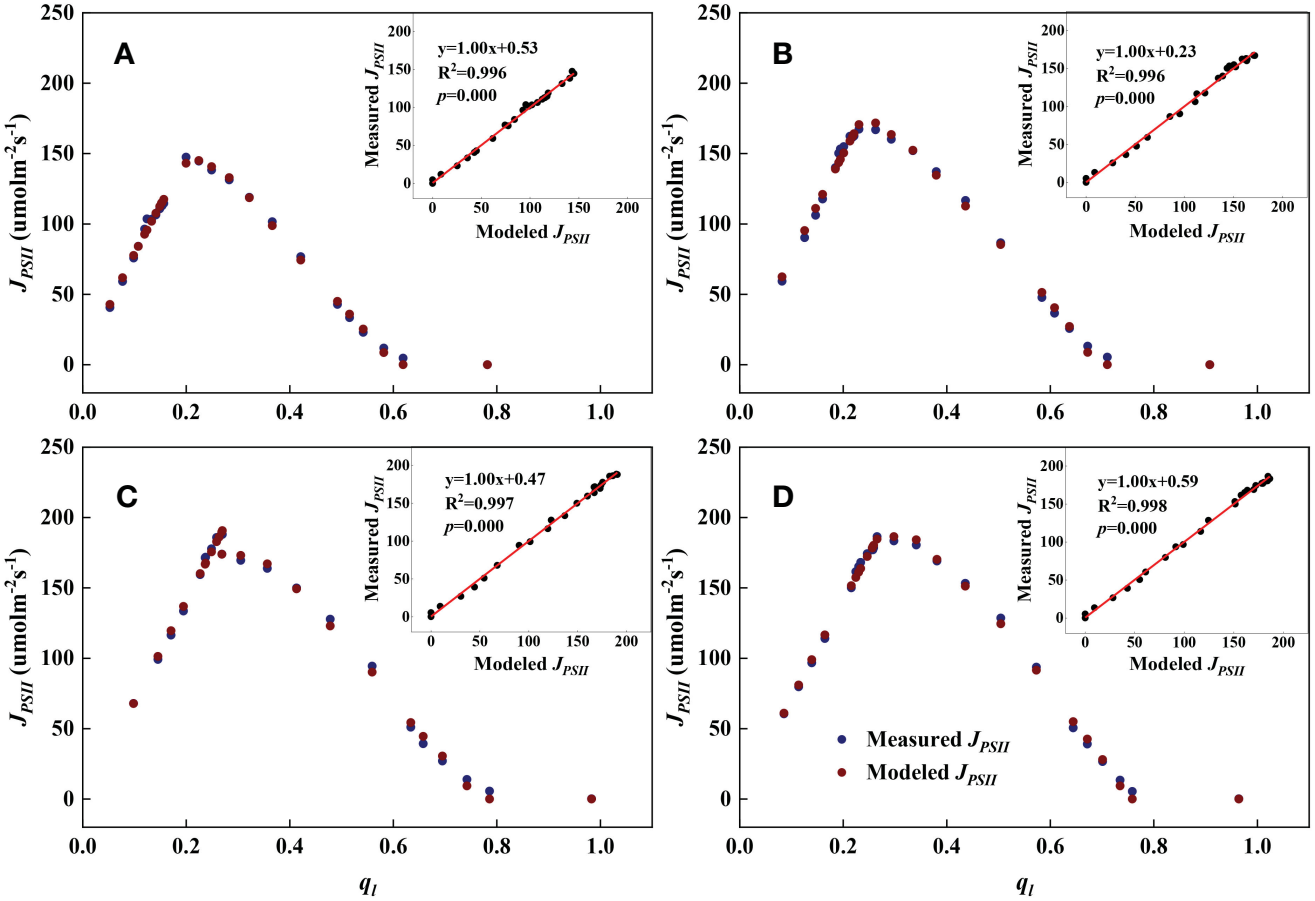
Examples demonstrating the performance of the steady-state photochemical model for predicting the linear electron transport rate (*J_PSII_*) as a function of the fraction of open PSII reaction centers ( $q_{l}$ ) at the flowering stage. Inset: Comparison of measured vs. modeled *J_PSII_*. **(A)**: Bottom; **(B)**: Lower middle; **(C)**: Upper middle; **(D)**: Top.

As shown in **Figures 1**, **2**, we found close agreement between the observed and modeled *J_PSII_* – *q* relationships at different growth stages and canopy heights and almost perfect correlations between modeled and measured *J_PSII_*. **Table 1** summarizes the test statistics for model performance. Our evaluation is consistent with that reported by Gu et al. (2023a), and indicated that the steady-state photochemical model performed well in reproducing the relationship between the linear electron transport rate and the fraction of open PSII reaction centers and that our use of the *J_PSII_* – *q* equation to infer redox parameters was warranted.

**Table 1:** The correlation between modeled and measured linear electron transport rate (*J_PSII_*).

Date	Position	Equation	R^2^	*p*
8 July		y=1.00x+0.34	0.987	0.000
17 July	Bottom	y=1.00x+0.29	0.996	0.000
	Top	y=1.00x+0.57	0.997	0.000
29 July	Bottom	y=1.00x+0.35	0.997	0.000
	Middle	y=1.00x+0.10	0.995	0.000
	Top	y=1.00x+0.17	0.997	0.000
11 August	Bottom	y=1.00x+0.53	0.996	0.000
	Lower middle	y=1.00x+0.23	0.995	0.000
	Upper middle	y=1.00x+0.47	0.997	0.000
	Top	y=1.00x+0.59	0.998	0.000
20 August	Bottom	y=1.02x-1.48	0.986	0.000
	Lower middle	y=1.00x+0.56	0.996	0.000
	Upper middle	y=0.99x+0.68	0.996	0.000
	Top	y=1.01x-0.74	0.995	0.000
7 September	Bottom	y=1.01x-0.49	0.995	0.000
	Lower middle	y=0.99x+0.60	0.998	0.000
	Upper middle	y=0.99x+0.82	0.998	0.000
	Top	y=0.99x+0.90	0.999	0.000
21 September	Bottom	y=1.00x-0.23	0.987	0.000
	Lower middle	y=1.00x-0.15	0.991	0.000
	Upper middle	y=1.00x+0.45	0.997	0.000
	Top	y=1.00x-0.60	0.997	0.000

### 3.2 Relationships between redox parameters and leaf characteristics

**Figure 3:**
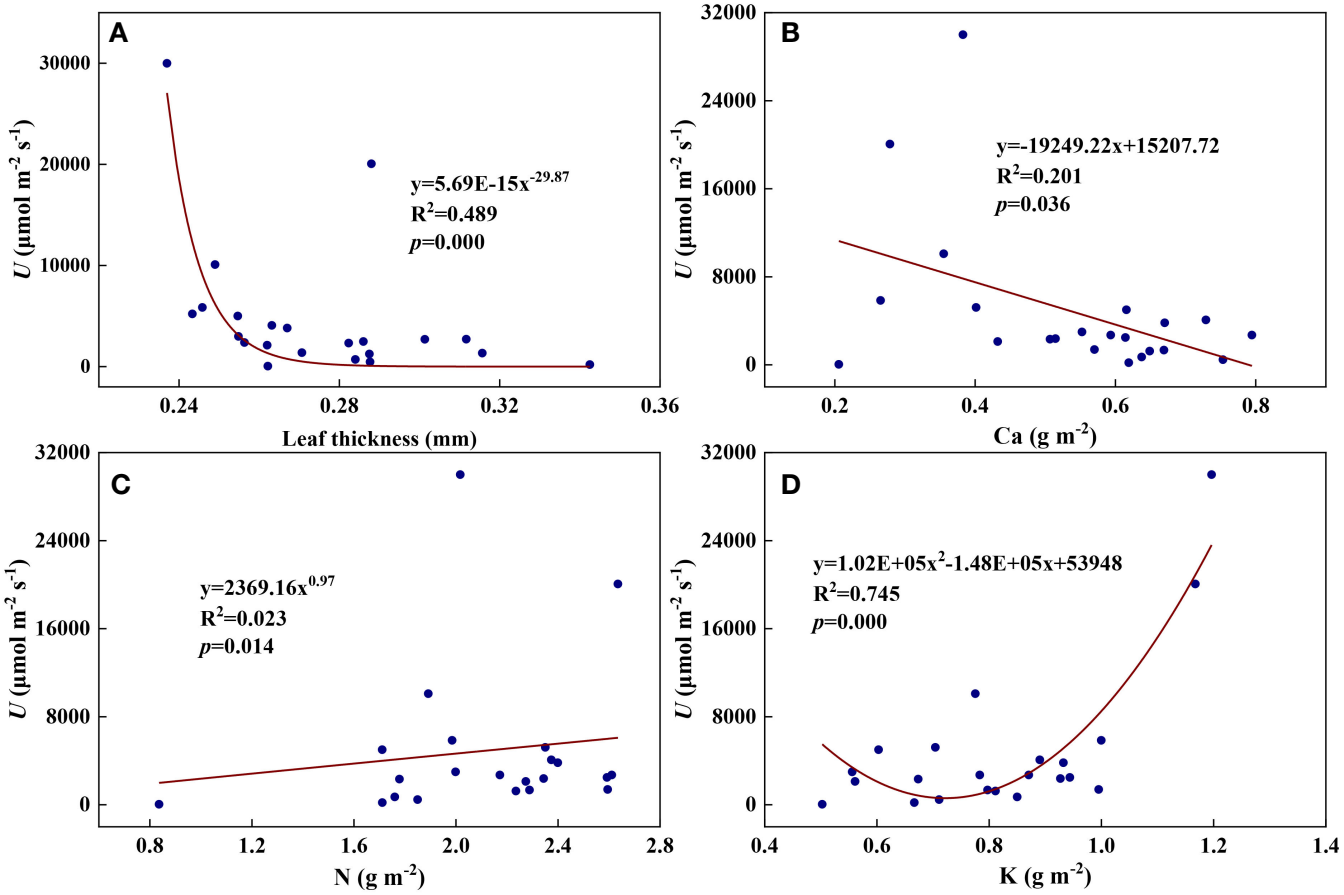
The relationships between the redox parameter *U* and leaf characteristics during summer maize growth. **(A)**: *U*-leaf thickness; **(B)**: *U*-Ca; **(C)**: *U*-N; **(D)**: *U*-K.

**Figure 4:**
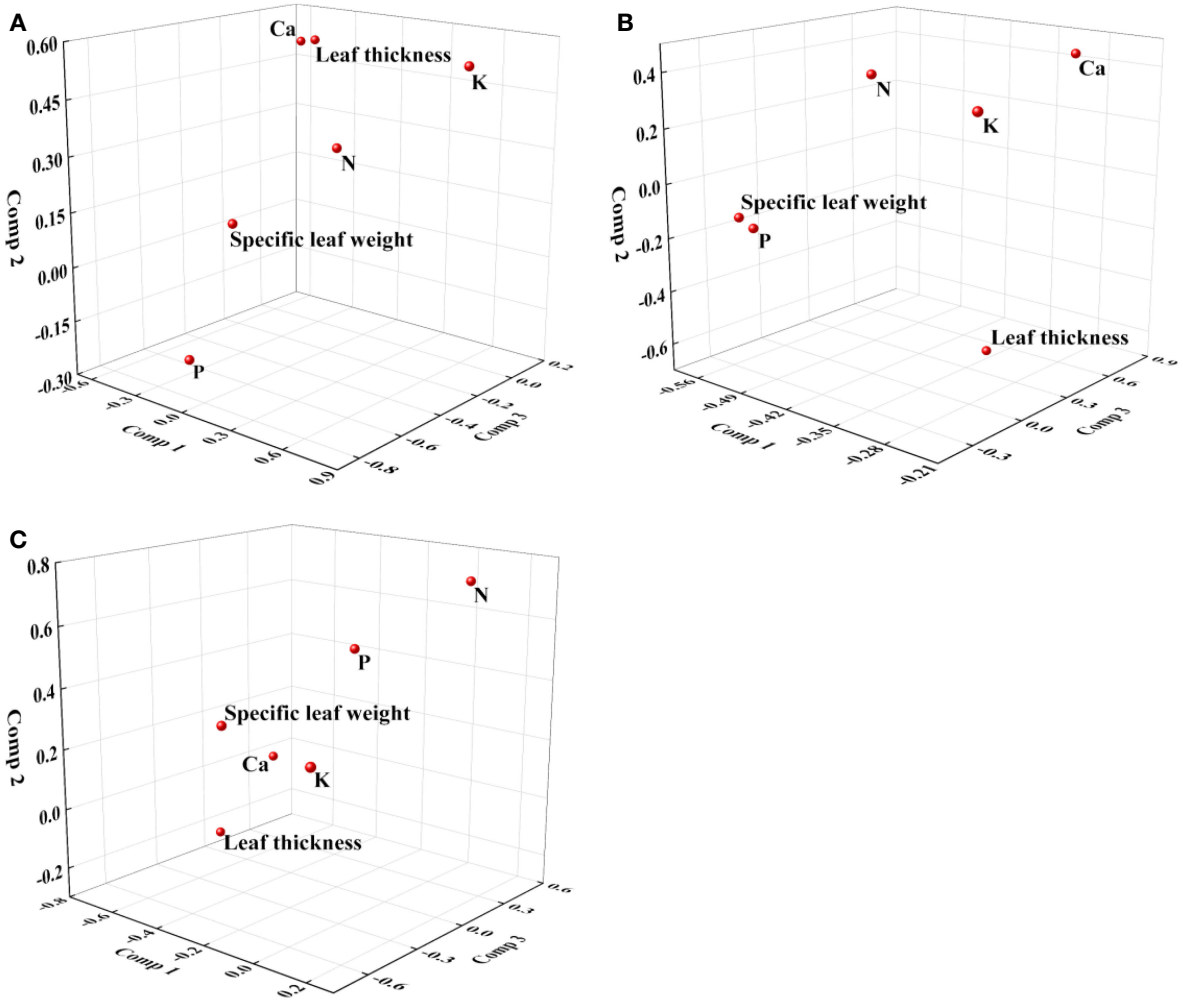
Loading plot of partial least squares regression for redox parameters and leaf characteristics. **(A)**: *U*; **(B)**: *R*_2_; **(C)**: *c_s_*.

**Figure 5:**
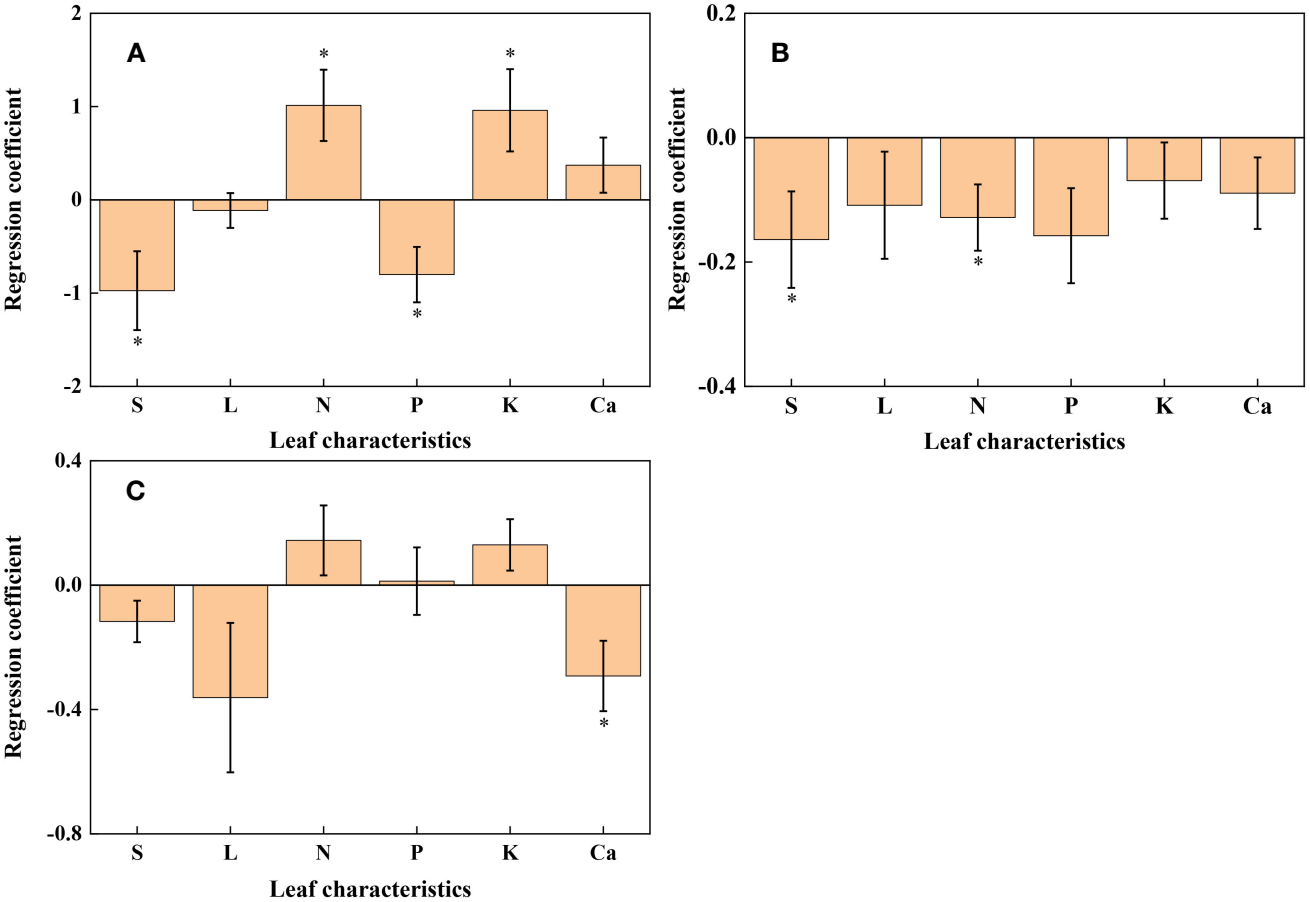
Histogram of regression coefficients of leaf characteristics affecting redox parameters. **(A)**: *U*; **(B)**: *R*_2_; **(C)**: *c_s_*. S: Specific leaf weight; L: Leaf thickness. ∗ Significant impact at *p*< 0.05.

**Figure 6:**
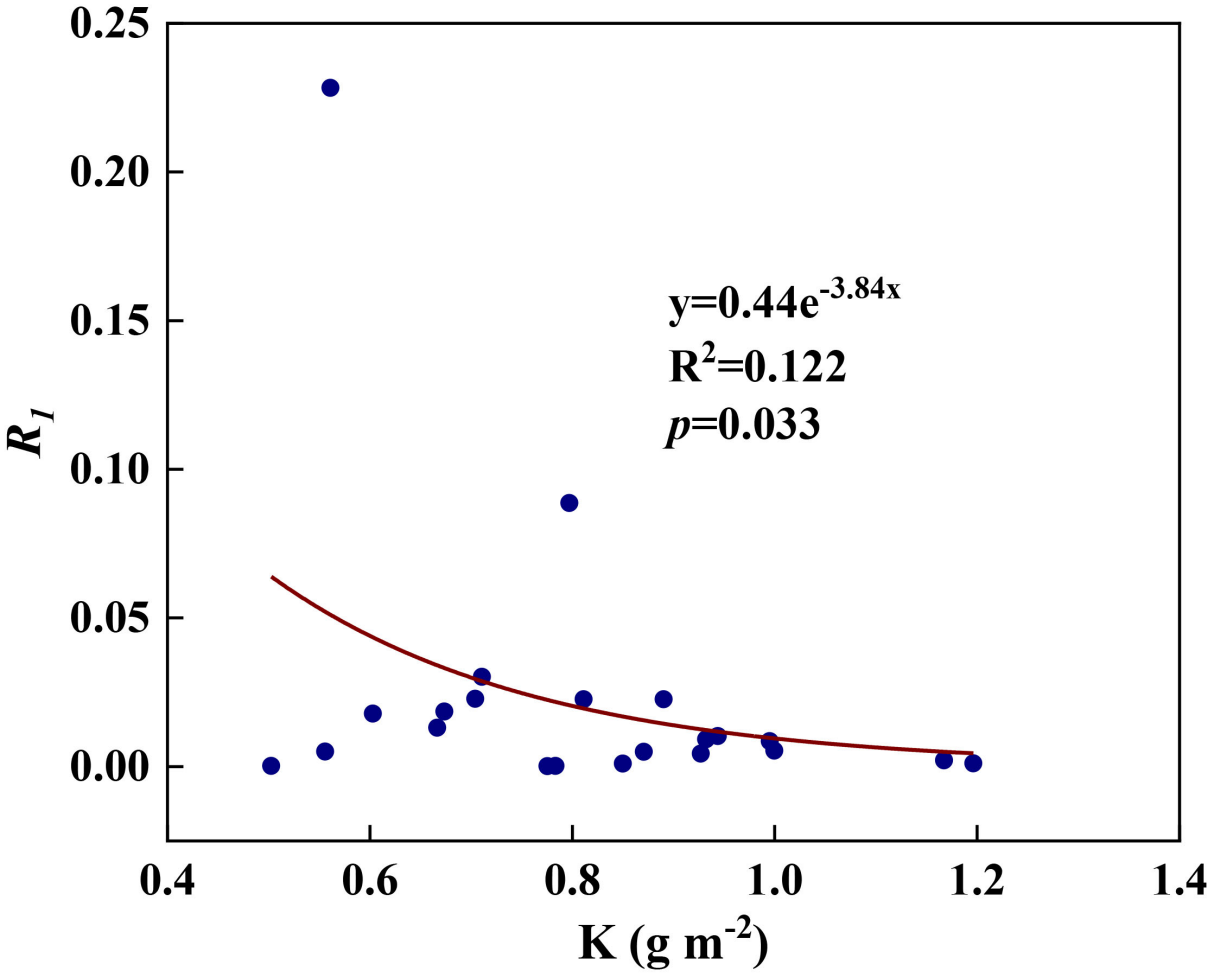
The relationships between redox parameter *R_1_* and leaf K content during summer maize growth.

**Figure 7:**
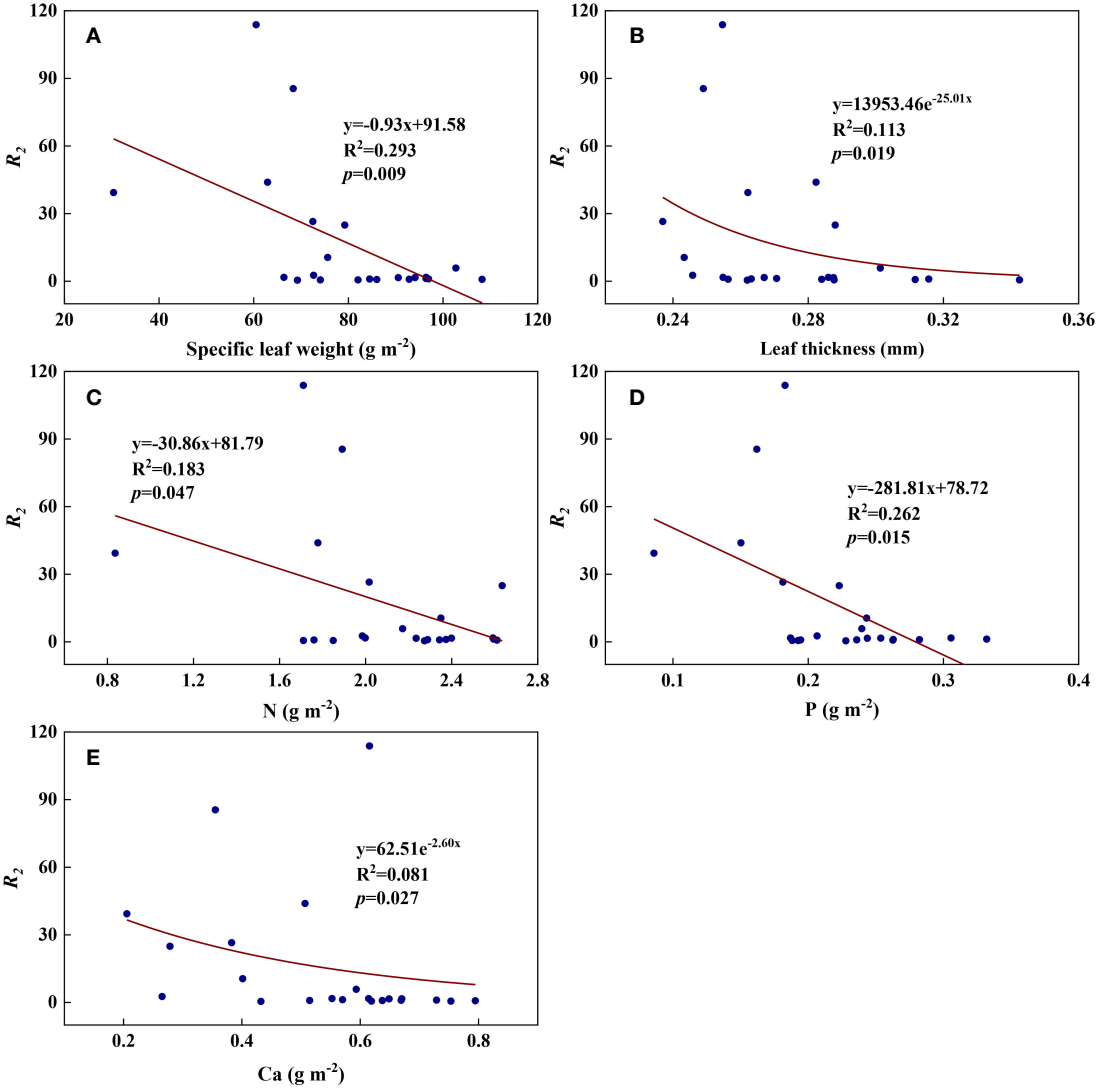
The relationships between redox parameter *R_2_* and leaf characteristics during summer maize growth. **(A)**: *R_2_*-specific leaf weight; **(B)**: *R_2_*-leaf thickness; **(C)**: *R_2_*-N; **(D)**: *R_2_*-P; **(E)**: *R_2_*-Ca.

**Figure 8:**
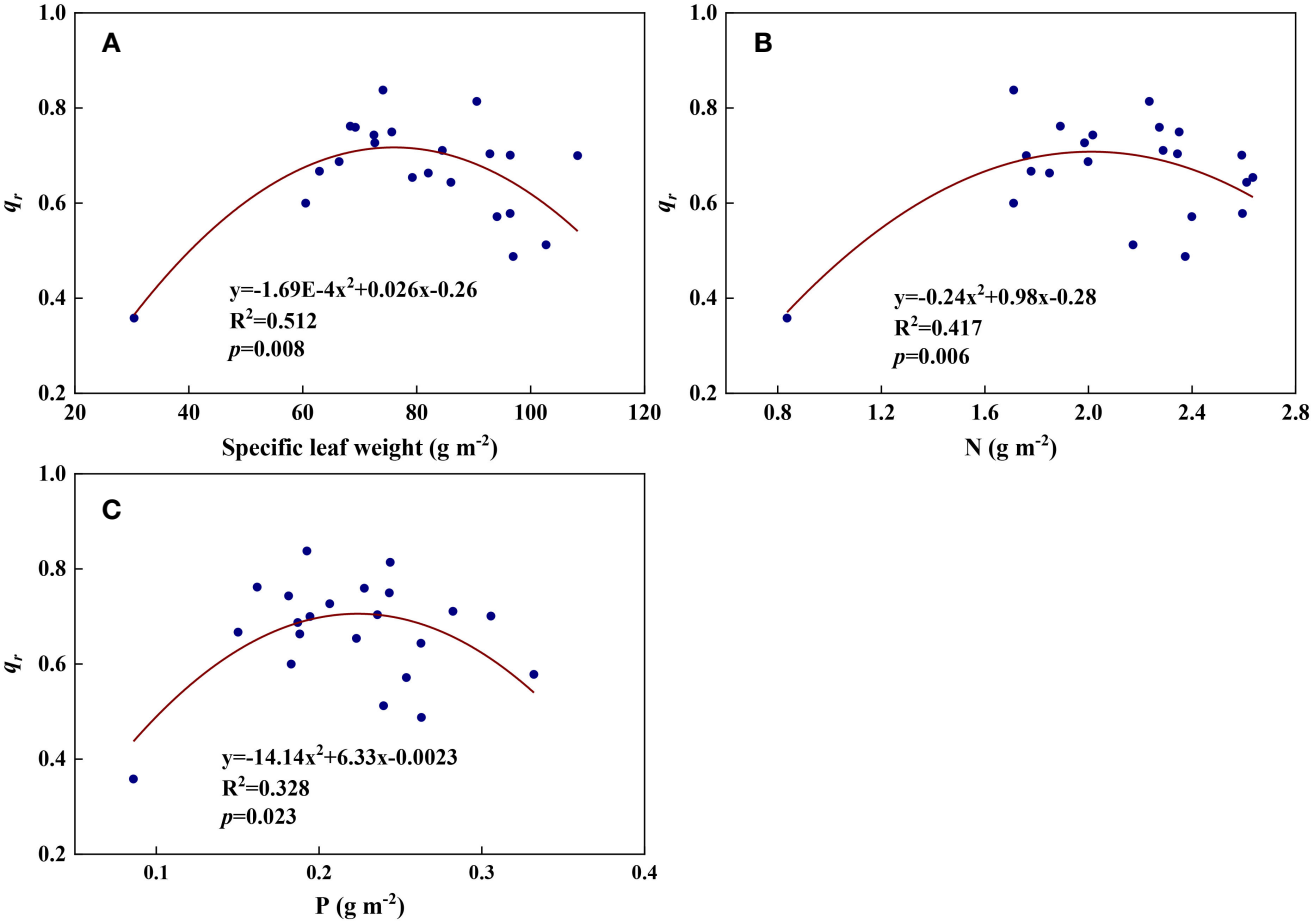
The relationships between redox parameter *q_r_* and leaf characteristics during summer maize growth. **(A)**: *q_r_*-specific leaf weight; **(B)**: *q_r_*-N; **(C)**: *q_r_*-P.

**Figure 9:**
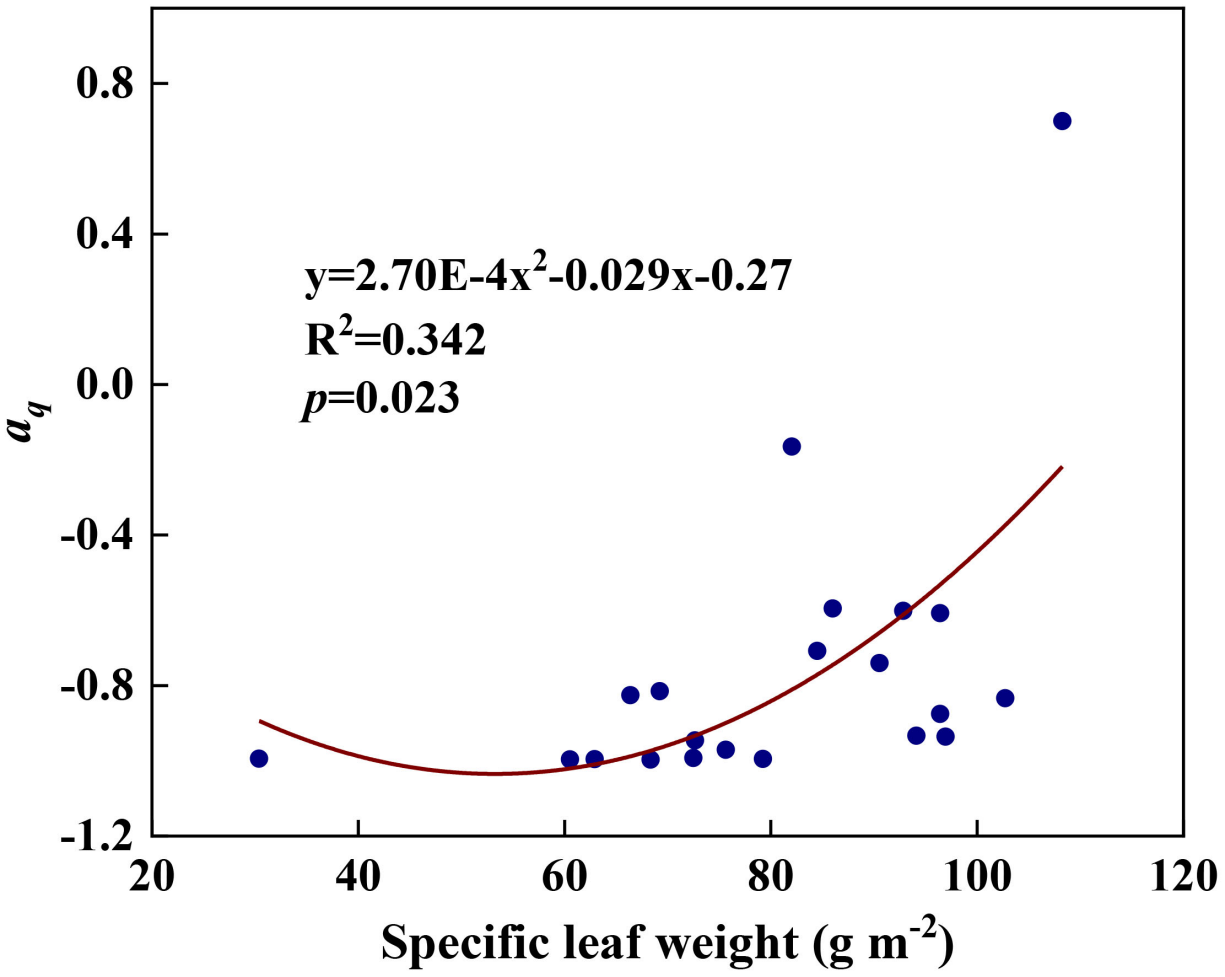
The relationships between redox parameter *a_q_* and specific leaf weight during summer maize growth.

**Figure 10:**
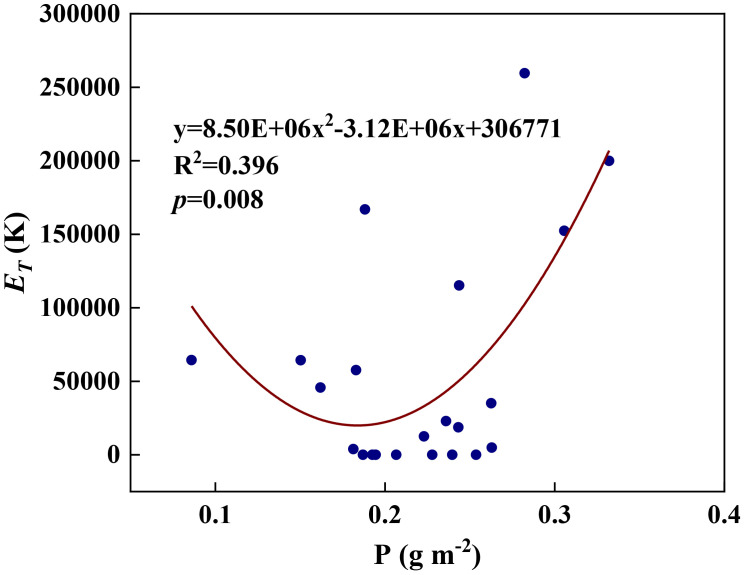
The relationships between redox parameter *E_T_* and leaf P content during summer maize growth.

**Figure 11:**
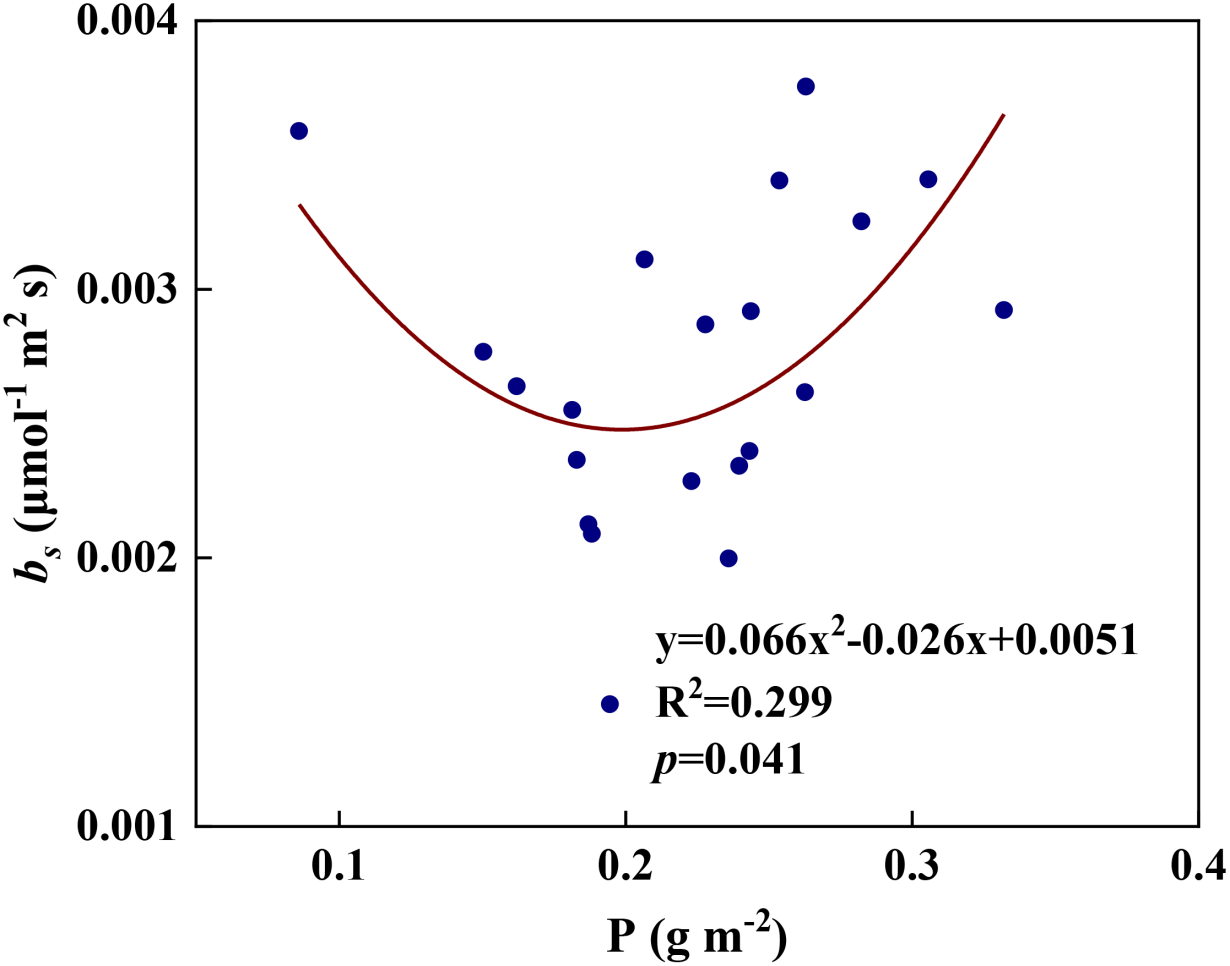
The relationships between redox parameter *b_s_* and leaf P content during summer maize growth.

**Figure 12:**
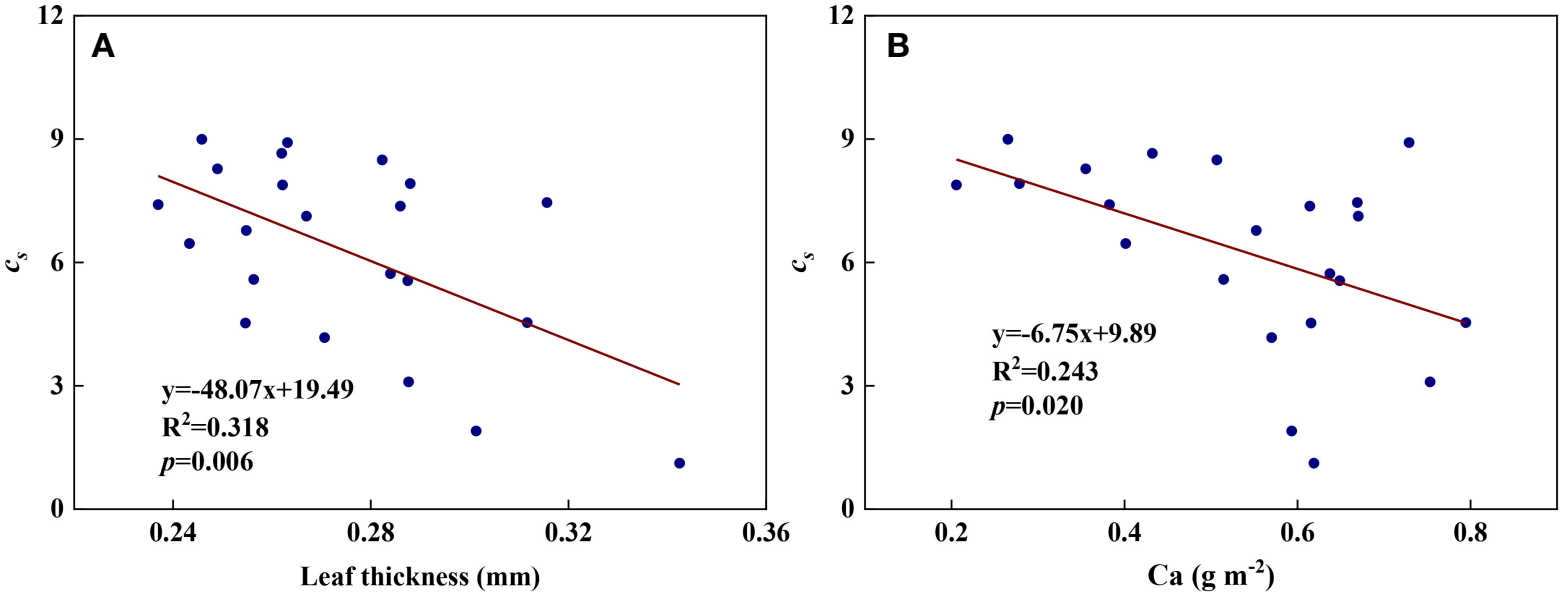
The relationships between redox parameter *c_s_* and leaf characteristics during summer maize growth. **(A)**: *c_s_*- leaf thickness; **(B)**: *c_s_*-Ca.

**Figure 13:**
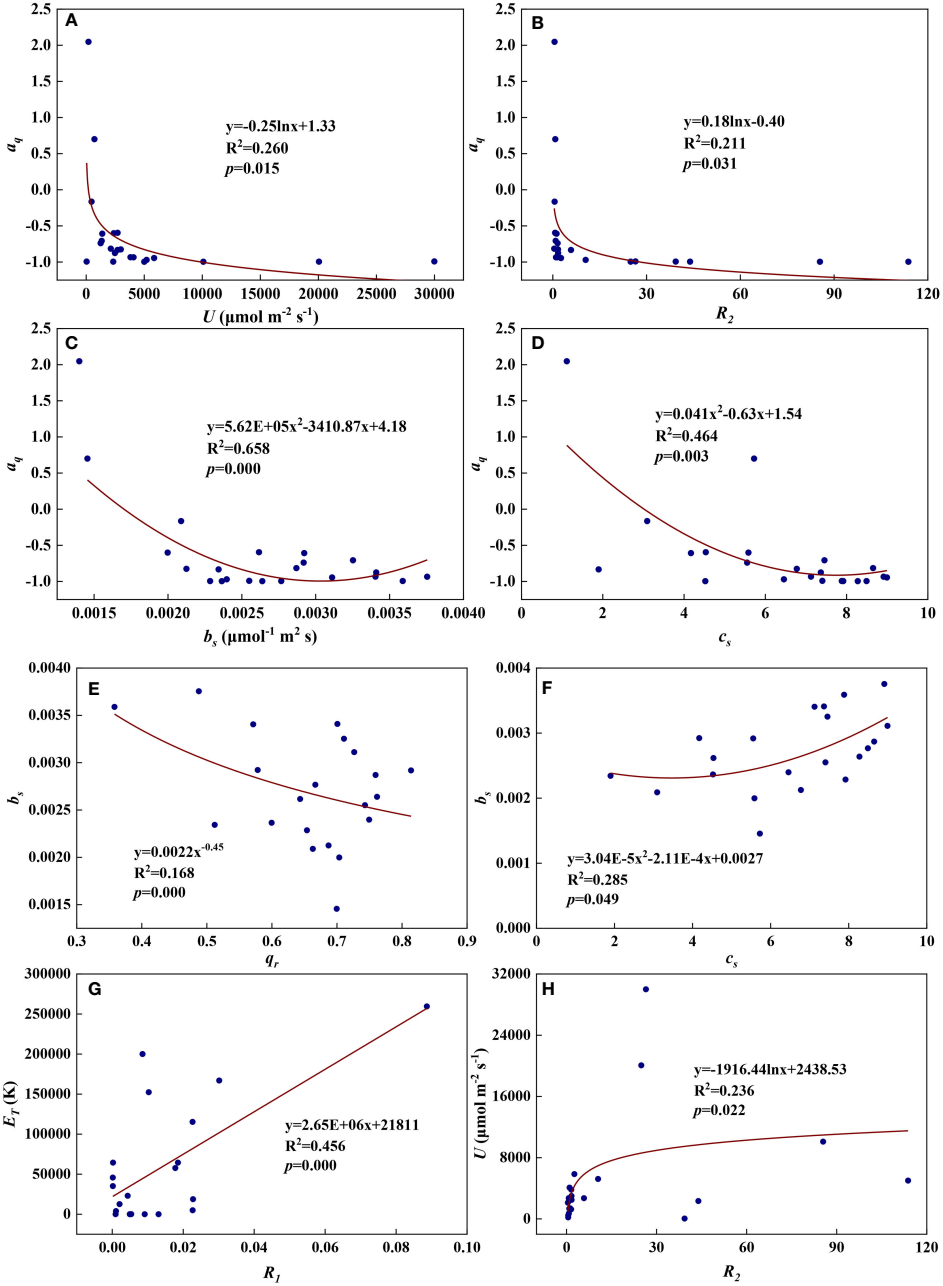
The relationships among redox parameters during summer maize growth. **(A)**: *a_q_*-*U*; **(B)**: *a_q_*-*R*_2_; **(C)**: *a_q_*-*b_s_*; **(D)**: *a_q_*-*c_s_*; **(E)**: *b_s_*-*q_r_*; **(F)**: *b_s_*-*c_s_*; **(G)**: *E_T_*-*R_1_*; **(H)**: *U*-*R_2_*.

We examined all 48 relationships between the eight redox parameters and six leaf characteristic parameters (8 × 6 = 48). 18 of the 48 relationships were statistically significant and are shown in **Figures 3**–**13**. *U* decreased with leaf thickness and Ca (**Figures 3A, B**) but increased with leaf N and K (**Figures 3C, D**). Power functions can reflect the relationships between *U* and leaf thickness (**Figure 3A**, R^2^ = 0.489, *p* = 0.000) and between *U* and N (**Figure 3C**, R^2^ = 0.023, *p* = 0.014), while a linear function and a quadratic polynomial can fit the relationship between *U* and Ca (**Figure 3B**, R^2^ = 0.201, *p* = 0.036), and that between *U* and K (**Figure 3D**, R^2^ = 0.745, *p* = 0.000), respectively. Partial least squares regression revealed that specific leaf weight (negative, *p*=0.031), N (positive, *p* = 0.015), P (negative, *p*=0.013), and K (positive, *p* = 0.041) significantly influenced *U* (R^2^ = 0.566, **Figures 4A**, **5A**). *R_1_* decreased with leaf K, and an exponential function can describe the relationship between them (**Figure 6**, R^2^ = 0.122, p = 0.033). *R_2_* decreased with specific leaf weight, leaf thickness, leaf N, P, and Ca (**Figures 7A–E**). Linear relationships were found between *R_2_* and specific leaf weight (**Figure 7A**, R^2^ = 0.293, *p* = 0.009), leaf N (**Figure 7C**, R^2^ = 0.183, *p* = 0.047), and P (**Figure 7D**, R^2^ = 0.262, *p* = 0.015), while exponential relationships were found between *R_2_* and leaf thickness (**Figure 7B**, R^2^ = 0.113, *p* = 0.019) and leaf Ca (**Figure 7E**, R^2^ = 0.081, *p* = 0.027). Partial least squares regression revealed that specific leaf weight (*p* = 0.047) and N (*p* = 0.025) were negatively related to *R_2_* (R^2^ = 0.073, **Figures 4B**, **5B**). *q_r_* appeared to be a peaked function of specific leaf weight, leaf N, and P, with both low and high values of these leaf characteristics lowering *q_r_* (**Figures 8A–C**). As a result, quadratic polynomial functions can describe the relationships between *q_r_* and specific leaf weight (**Figure 8A**, R^2^ = 0.512, *p* = 0.008), between *q_r_* and N (**Figure 8B**, R^2^ = 0.417, *p* = 0.006), and between *q_r_* and P (**Figure 8C**, R^2^ = 0.328, *p* = 0.023). *a_q_* increased with specific leaf weight (**Figure 9**), and *E_T_* and *b_s_* increased with leaf P (**Figures 10**, **11**). Quadratic polynomial functions can model the relationships between *a_q_* and specific leaf weight (**Figure 9**, R^2^ = 0.342, *p* = 0.023), between *E_T_* and P (**Figure 10**, R^2^ = 0.396, *p* = 0.008), and between *b_s_* and P (**Figure 11**, R^2^ = 0.299, *p* = 0.041). *c_s_* decreased with leaf thickness and leaf Ca (**Figures 12A, B**). Linear functions can fit the relationships between *c_s_* and leaf thickness (**Figure 12A**, R^2^ = 0.318, *p* = 0.006) and between *c_s_* and Ca (**Figure 12B**, R^2^ = 0.243, *p* = 0.020). Partial least squares regression revealed that Ca was negatively correlated with *c_s_* (R^2^ = 0.079, *p* = 0.017, **Figures 4C**, **5C**). As shown in **Figure S1**, the remaining 30 of the 48 relationships are highly scattered and are not statistically significant; these plots will not be discussed here.

### 3.3 Relationships between redox parameters

We examined all 28 relationships between any two of the eight redox parameters (8×7/2 = 28). Eight of the 28 relationships were statistically significant and are shown in **Figure 12**. *a_q_* decreased with *U*, *R_2_*, *b_s_*, and *c_s_* (**Figures 13A–D**). Logarithmic functions can describe the relationships between *a_q_* and *U* (**Figure 13A**, R^2^ = 0.260, *p* = 0.015) and between *a_q_* and *R_2_* (**Figure 13B**, R^2 =^ 0.211, *p* = 0.031), while quadratic polynomial functions can describe the relationships between *a_q_* and *b_s_* (**Figure 13C**, R^2^ = 0.658, *p* = 0.000) and between *a_q_* and *c_s_* (**Figure 13D**, R^2^ = 0.464, *p* = 0.003). *b_s_* decreased with *q_r_* but increased with *c_s_* (**Figures 13E, F**). A power function can fit the relationship between *b_s_* and *q_r_* (**Figure 13E**, R^2^ = 0.168, *p* = 0.000), while a quadratic polynomial function can model the relationship between *b_s_* and *c_s_* (**Figure 13F**, R^2^ = 0.285, *p* = 0.049). *E_T_* increased with *R_1_* and a linear function fit the relationship between them well (**Figure 13G**, R^2^ = 0.456, *p* = 0.000). *U* increased with *R_2_* and a logarithmic function can describe the relationship between them (**Figure 13H**, R^2^ = 0.236, *p* = 0.022). As shown in **Figure S2**, the remaining 20 of the 28 relationships did not reach statistical significance.

### 3.4 Temporal variations of redox parameters

**Figure 14:**
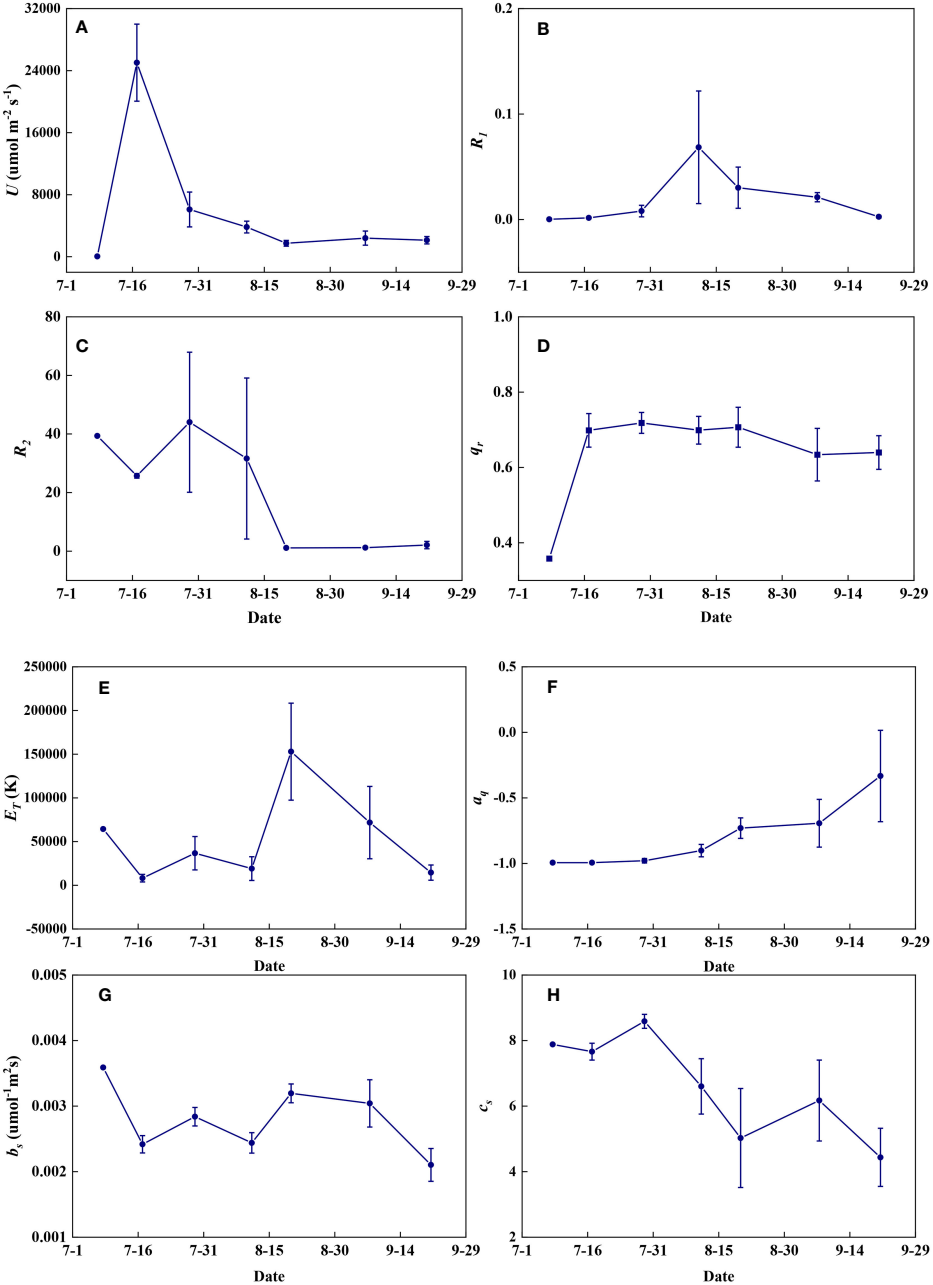
The temporal variations of redox parameters during summer maize growth. **(A)**: *U*; **(B)**: *R_1_*; **(C)**: *R_2_*; **(D)**: *q_r_*; **(E)**: *E_T_*; **(F)**: *a_q_*; **(G)**: *b_s_*; **(H)**: *c_s_*.

Redox parameters varied significantly from seedling to maturity. *U* increased significantly from the seedling to the jointing stage and then decreased rapidly to the filling stage but remained almost constant at the maturity stage (**Figure 14A**). *R_1_* increased from the seedling to the flowering stage and then decreased with grain maturation (**Figure 14B**). *R_2_* fluctuated at high levels until the flowering stage and then remained nearly constant during the maturity stages (**Figure 14C**). *q_r_* increased steeply at the jointing stage and then fluctuated at relatively high levels until maturity (**Figure 14D**). *E_T_* fluctuated at low levels until the flowering period and increased steeply at the filling stage but decreased with grain maturation (**Figure 14E**). *a_q_* remained nearly constant until the flowering period and then gradually increased during the maturity stages (**Figure 14F**). *b_s_* was highest in seedlings, and remained relatively constant throughout the growth period, but decreased at the end of late maturation (**Figure 14G**). *c_s_* remained nearly constant until the flowering period and then decreased in a fluctuating way until maturity (**Figure 14H**).

### 3.5 Temporal variations of leaf characteristics

**Figure 15:**
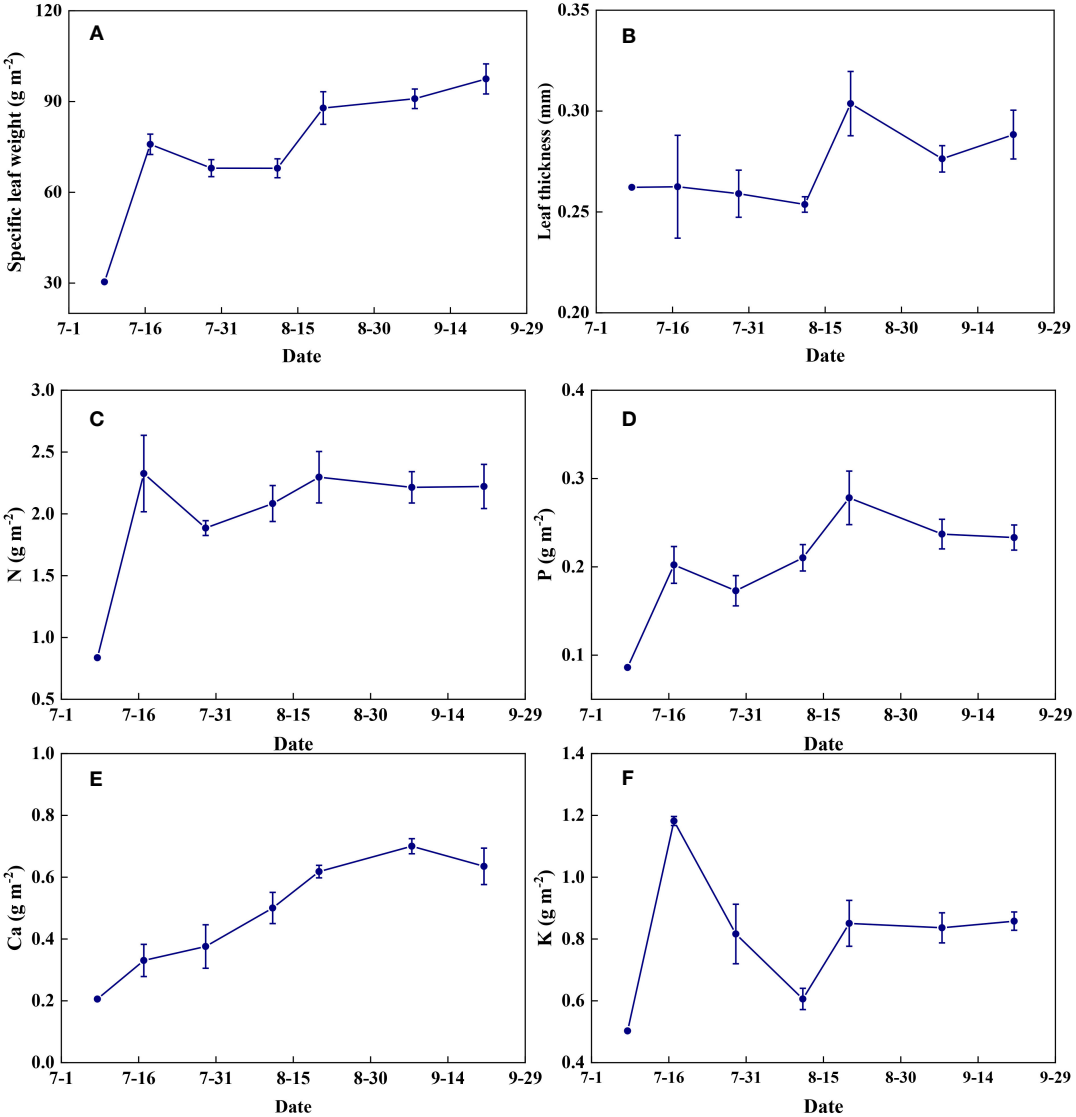
The temporal variations of leaf characteristics during summer maize growth. **(A)**: specific leaf weight; **(B)**: leaf thickness; **(C)**: N; **(D)**: P; **(E)**: Ca; **(F)**: K.

Leaf physical traits and nutrient contents varied dynamically between different growth stages (**Figure 15**). Specific leaf weight increased gradually with the growth of summer maize (**Figure 15A**). Leaf thickness remained relatively stable before flowering and increased thereafter (**Figure 15B**). Leaf N content increased significantly from seedling to jointing stages and then remained almost constant throughout the growth period (**Figure 15C**). Leaf P and Ca contents increased with the growth of summer maize and decreased at the late stages of grain maturation (**Figures 15D, E**). Leaf K content increased rapidly at the jointing stage, decreased at the flowering stage, and then remained relatively constant at the filling stage until maturity (**Figure 15F**).

## 4 Discussion and conclusion

Our study found that there is considerable variation in photochemical redox parameters inferred from PAM fluorometry measurements in maize. Part of these variations may be due to measurement noise and a lack of data constraints on parameter optimization (*i.e.*, overfitting), while statistically significant relationships between these parameters and independently measured leaf macronutrient content and morphology, as well as their systematic variations with developmental stage, suggest that at least part of these variations may be due to changes in the structure of the ETC. All parameters in the photochemical model of electron transport are composite parameters that are influenced by multiple components of the ETC. For example, *U* is the main parameter that determines the capacity of the ETC. It is the product of the rate constant for the oxidation of PQH_2_ by the RieskeFeS protein of Cyt and the abundances of the PQ and Cyt pools. Although PQ does not contain nitrogen, Cyt contains nitrogen in its hemes (Tikhonov, 2023). Therefore, the positive correlation between *U* and leaf nitrogen probably reflects the higher abundance of Cyt at higher leaf nitrogen contents. The positive correlation between *U* and leaf nitrogen contributes to the balance between electron transport and CO_2_ assimilation, which is catalyzed by Rubisco, a dominant sink of leaf nitrogen (Evans and Clarke, 2019). Potassium is not involved in the structure of organic macromolecules. However, it is the most important inorganic osmotic ion in plant cells and plays an important role in controlling stomatal conductance and in maintaining the structural integrity of granal thylakoids, where photosynthetic macromolecules are located (Tränkner et al, 2018), which likely explains the positive correlation between *U* and potassium.

The resistance parameters *R_1_* and *R_2_* are given by *R_1_* = $\frac{r_{r}}{r_{d}}$ and $R_{2}=\frac{u}{r_{d}}\times \frac{N_{cyt_{T}}}{N_{PSII}}$ , respectively. *r_d_* and *r_r_* are the second-order rate constants for the forward and reverse reactions between the reduced acceptor of PSII and PQ, respectively, while *u* is the second-order rate constant for the oxidation of PQH_2_ by the RieskeFeS protein of Cyt. Thus, *R_1_* is inversely related to the efficiency of PQ to take away electrons from PSII, and *R_2_* is inversely related to the capacity of PSII to supply electrons relative to the capacity of Cyt to oxidize PQH_2_. The smaller the values of *R_1_* and *R_2_*, the higher the efficiency of the ETC. Thus, the decrease of *R_1_* with potassium and *R_2_* with nitrogen and phosphorus is consistent with the expected impact of these nutrients on electron transport.

A significant fraction of PSII reaction centers may be Q_B_-nonreducing (Tomek et al., 2003; Schansker and Strasser, 2005; Vredenberg et al., 2006; Vredenberg, 2011), which can result in *q_r_* being significantly less than 1. Currently, little is known about why such reaction centers exist at all and what their photochemical functions may be. The significant correlation between *q_r_* and leaf nitrogen may suggest that the fraction of Q_B_-nonreducing centers is affected by leaf nitrogen content.

Any factors that affect the relative abundances of PSII and Cyt can affect the value of the parameter *a_q_*. A negative *a_q_* indicates that there is a greater constraint on Cyt than on PSII for electron transport, whereas a positive *a_q_* indicates the opposite (Gu et al., 2023a). The increase in *a_q_* with specific leaf weight likely indicates that the relative abundance of PSII vs. Cyt in a leaf depends on leaf-specific weight. Variations in *b_s_* and *c_s_* may reflect systematic changes in thylakoid structure. Such changes may include the number of grana per thylakoid, the height of a granum, and the density of ion channels, which affect ion exchange between the lumen and stroma and, therefore, osmotic water fluxes. These changes can affect the degree and speed of thylakoid swelling/shrinking and, therefore, the values of *b_s_* and *c_s_*. Variations in *E_T_* can be caused by changes in the Gibbs free energy of activation (Gu et al., 2023a), which in turn can be caused by changes in the reduction potentials of redox reactions (Silverstein, 2012; Bostick et al., 2018). Thus, the variation of *E_T_* with leaf phosphorus content may indicate that the latter affects the reduction potentials of redox reactions along the ETC.

Currently, it is difficult to provide an in-depth discussion of all the relationships reported in this study. There are very few previous studies that have investigated how the structures and redox reactions of the ETC may be affected by nutrient contents and plant developmental stages, and thus may provide guidance for our analysis. Nevertheless, our study provides conclusive evidence that the structural properties of the electron transport chain are not static and may vary with plant growth conditions and developmental stages, allowing the photophysical, photochemical, and biochemical reactions to be balanced on seasonal time scales. Our findings also show that it may be possible to parameterize photochemical parameters of electron transport with leaf nutrient content and morphological properties for large-scale modeling applications.

## Author’s note

This manuscript was co-authored by UT-Battelle, LLC under contract number DE-AC05-00OR22725 with the U.S. Department of Energy. The publisher, by accepting the article for publication, acknowledges that the United States Government retains a non-exclusive, paid-up, irrevocable, worldwide license to publish or reproduce, or allow others to do so, the published form of this manuscript for the purposes of the United States Government. The Department of Energy will provide public access to these results of federally sponsored research in accordance with the DOE Public Access Plan (http://energy.gov/downloads/doe-public-access-plan).

## Data availability statement

The raw data supporting the conclusions of this article will be made available by the authors, without undue reservation.

## Author contributions

XL: Investigation, Formal analysis, Writing – original draft. YQ: Investigation, Writing – review & editing. WZ: Investigation, Writing – review & editing. WD: Supervision, Writing – review & editing. LG: Methodology, Writing – original draft.
