# Supplementary material for: Determinants of photochemical characteristics of the photosynthetic electron transport chain of maize
Source: Front Plant Sci. 2023 Nov 20;14:1279963. doi: 10.3389/fpls.2023.1279963 (PMC10694277; doi:10.3389/fpls.2023.1279963)
Supplement: Supplementary file 1 [file DataSheet_1.docx]

Figure S1 The relationships between redox parameters and leaf characteristics during summer maize growth. (A1): *U*-specific leaf weight; (B1): *U*-P; (C1): *R_1_*-specific leaf weight; (D1): *R_1_*-leaf thickness; (E1): *R_1_*-N; (F1): *R_1_*-P; (G1): *R_1_*-Ca; (H1): *R_2_*-K; (I1): *q_r_*-leaf thickness; (J1): *q_r_*-K; (K1): *q_r_*-Ca; (L1): *a_q_*-leaf thickness; (M1): *a_q_*-N; (N1): *a_q_*-P; (O1): *a_q_*-K; (P1): *a_q_*-Ca; (Q1): *E_T_*-specific leaf weight; (R1): *E_T_*-leaf thickness; (S1): *E_T_*-N; (T1): *E_T_*-K; (U1): *E_T_*-Ca; (V1): *b_s_*-specific leaf weight; (W1): *b_s_*-leaf thickness; (X1): *b_s_*-N; (Y1): *b_s_*-K; (Z1): *b_s_*-Ca; (A2): *c_s_*-specific leaf weight; (B2): *c_s_*-N; (C2): *c_s_*-P; (D2): *c_s_*-K.

Figure S2 The relationships among redox parameters during summer maize growth. (A): *a_q_*-*R_1_*; (B): *a_q_*-*q_r_*; (C): *a_q_*-*E_T_*; (D): *b_s_*-*U*; (E): *b_s_*-*R_1_*; (F): *b_s_*-*R_2_*; (G): *b_s_*-*E_T_*; (H): *E_T_*-*U*; (I): *E_T_*-*R_2_*; (J): *E_T_*-*q_r_*; (K): *E_T_* -*c_s_*; (L): *U*-*R_1_*; (M): *U*-*q_r_*; (N): *U*-*c_s_*; (O): *R_1_*-*R_2_*; (P): *R_1_*-*q_r_*; (Q): *R_1_*-*c_s_*; (R): *R_2_*-*q_r_*; (S): *R_2_*-*c_s_*; (T): *q_r_*-*c_s_*.
